# Abnormal Splicing Events due to Loss of Nuclear Function of TDP-43: Pathophysiology and Perspectives

**DOI:** 10.31662/jmaj.2024-0038

**Published:** 2024-06-17

**Authors:** Yuka Koike

**Affiliations:** 1 Department of Molecular Neuroscience, Brain Research Institute, Niigata University, Niigata, Japan; 2 Department of Neuroscience, Mayo Clinic Florida, Florida, USA

**Keywords:** ALS/FTD, TDP-43, Loss of function, Cryptic exon, TDP-43 autoregulatory mechanism

## Abstract

Amyotrophic lateral sclerosis (ALS) and frontotemporal dementia (FTD) are neurodegenerative diseases with a progressive and fatal course. They are often comorbid and share the same molecular spectrum. Their key pathological features are the formation of the aggregation of TDP-43, an RNA-binding protein, in the cytoplasm and its depletion from the nucleus in the central nervous system. In the nucleus, TDP-43 regulates several aspects of RNA metabolism, ranging from RNA transcription and alternative splicing to RNA transport. Suppressing the aberrant splicing events during RNA processing is one of the significant functions of TDP-43. This function is impaired when TDP-43 becomes depleted from the nucleus. Several critical cryptic splicing targets of TDP-43 have recently emerged, such as *STMN2*, *UNC13A*, and others. *UNC13A* is an important ALS/FTD risk gene, and the genetic variations, single nucleotide polymorphisms, cause disease via the increased susceptibility for cryptic exon inclusion under the TDP-43 dysfunction. Moreover, TDP-43 has an autoregulatory mechanism that regulates the splicing of its mRNA (*TARDBP* mRNA) in the healthy state. This study provides recent findings on the splicing regulatory function of TDP-43 and discusses the prospects of using these aberrant splicing events as efficient biomarkers.

## 1. Introduction: Amyotrophic Lateral Sclerosis and Frontotemporal Dementia as TDP-43 Proteinopathy

Amyotrophic lateral sclerosis (ALS) is an adult-onset neurodegenerative disease that is characterized by motor neuron-specific degeneration, which causes progressive muscle weakness, fasciculation, and atrophy, leading to respiratory failure ^(1)^. Fifteen percent of patients with ALS have concomitant frontotemporal dementia (FTD) with cognitive, behavioral, and language deficits ^(2)^.

After the discovery of TDP-43 pathology associated with both ALS and FTD in 2006, these two diseases are firmly placed on a spectrum with similar underlying molecular mechanisms ^(3), (4)^. TDP-43 is an RNA-binding protein encoded by the *TARDBP* gene generally localized in the nucleus ^(1), (2)^. In the nucleus, TDP-43 usually regulates multiple aspects of RNA processing, including pre-messenger RNA (pre-mRNA) splicing ^(5)^. Nuclear depletion and cytoplasmic aggregation of TDP-43 are key pathological features in more than 97% of ALS cases and nearly 50% of cases with FTD (FTLD-TDP) ^(1), (2)^.

## 2. The Role of TDP-43, as a Repressor of “Cryptic Exon” Inclusion

**Figure 1.**
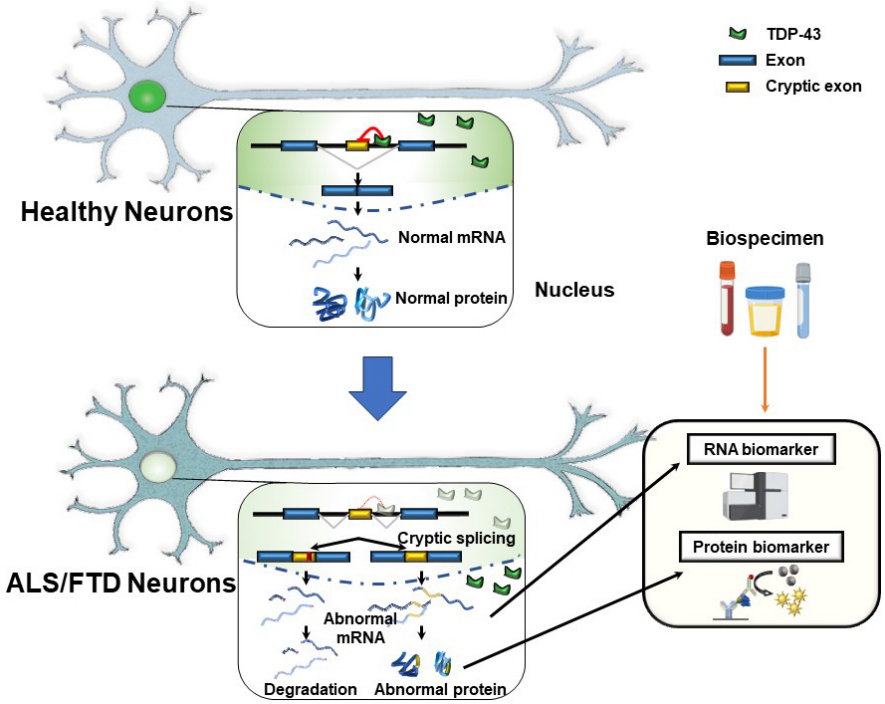
The mechanism regulating the splicing events by TDP-43 in the nucleus and cryptic splicing events as potential biomarkers. In healthy neurons, TDP-43 localizes to the nucleus. Cryptic exons are within introns that should not be included in the mature mRNA following splicing. Nuclear TDP-43 represses the inclusion of cryptic exons. Nuclear depletion of TDP-43 in ALS/FTD neurons leads to cryptic splicing, generating abnormal mRNA and abnormal protein. Cryptic splicing events can function as novel biomarker candidates, which reflects TDP-43 dysfunction. These biomarkers could be RNA- and protein-based. This figure modified from Reference 8.

Suppressing cryptic exons during pre-mRNA splicing is one of the critical functions of TDP-43 in the nucleus ^(6), (7)^. Exons, which code for proteins, are spliced together in normal splicing, while introns, the intervening sequences, are spliced out. However, some genes have sequences within introns that mimic exons and can be incorrectly included in the mRNA. The “cryptic exons” can disrupt the reading frame of the proteins. Accordingly, including cryptic exons destabilizes the mRNAs, leading to their degradation or coding aberrant peptides. TDP-43 is crucial in inhibiting these cryptic exons from slipping into mRNAs. There are binding sites for TDP-43 around the cryptic exon sequences. When TDP-43 is functional in the nucleus, the cryptic exons are kept out of mRNA (Figure 1) ^(7), (8)^. However, when the TDP-43 function is depleted in the nucleus, these cryptic exons slip into the mRNAs of many different genes ^(7), (8)^. The discovery that TDP-43 represses cryptic exons provides exciting insights for explaining the molecular mechanisms underlying ALS and FTD. We can expect the development of disease-specific biomarkers and effective therapeutic targets based on the pathogenesis. However, it remains unclear which cryptic splicing events are critical for TDP-43 proteinopathy.

## 3. TDP-43-dependent Cryptic Splicing Event in *Stathmin2* Associates with Neurodegeneration in ALS/FTD

In 2019, two independent studies uncovered an important human cryptic splicing target of TDP-43―*stathmin 2* (*STMN2*), which encodes a protein regulating microtubule stability in neurons ^(9), (10)^. RNA sequencing data from human neuronal cells with TDP-43 depleted were analyzed. The analysis found that the *STMN2* gene harbors a cryptic exon (the so-called exon 2a) that is usually not included in mature *STMN2* mRNA. Since the first intron of *STMN2* contains a TDP-43 binding site, TDP-43 typically suppresses, including cryptic exon. When TDP-43 is depleted in the nucleus, exon 2a is incorporated into mature mRNA. This exon harbors a stop codon and a premature polyadenylation signal, producing truncated *STMN2* mRNA ^(9), (10)^. Abnormal splicing variants and reduced STMN2 protein levels are significant features of familial and sporadic ALS cases with TDP-43 pathology. This cryptic splicing event in *STMN2* represents critical neuronal impairments caused by TDP-43 dysfunction. Both teams found that upregulating STMN2 can rescue the defects of axonal regeneration due to TDP-43 depletion in human iPSC-derived neurons ^(9), (10)^. These facts indicate that the dysregulation of the *STMN2* splicing partially causes TDP-43-dependent neurodegeneration. Moreover, *STMN2* cryptic exon inclusion plays a role in the pathogenesis of FTD ^(11)^. Truncated *STMN2* RNA is elevated in the postmortem brain tissues from FTLD-TDP-43 cases but not in controls or cases with progressive supranuclear palsy, a different neurodegenerative disease without TDP-43 pathology. Of interest, truncated *STMN2* levels correlate with phosphorylated TDP-43 protein levels ^(11)^. We speculate that *STMN2* cryptic exon inclusion may be prior to the formation of TDP-43 in the neurons, even without TDP-43 nuclear depletion. Thus, TDP-43-dependent cryptic splicing events in ALS and FTD may allow us to detect the earliest events of TDP-43 pathology. The breakthrough related to the *STMN2* cryptic splicing event can provide a clue to explain the pathogenesis of TDP-43 proteinopathy.

## 4. TDP-43-dependent Cryptic Splicing Event in *UNC13A*, a Key ALS/FTD Risk Gene

**Figure 2.**
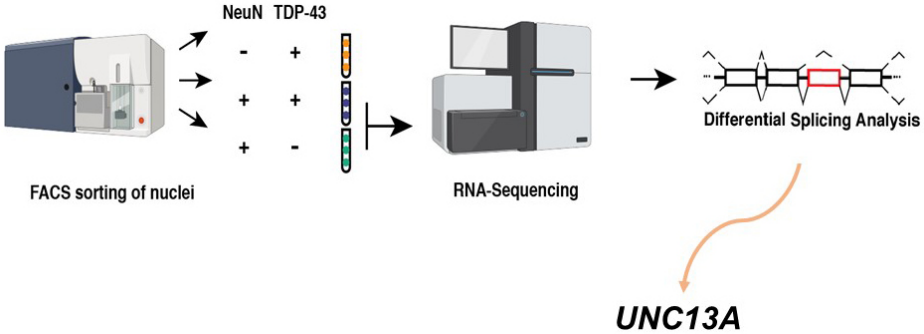
Detection of *UNC13A* cryptic exon. The identification process of the *UNC13A* abnormal splicing variant, including cryptic exon in TDP-43-negative neuronal nuclei using FACS. (-)/(+) in the figure means negative/positive NeuN (Neuronal-Nuclei, neuronal marker) antibody or TDP-43 antibody signal, respectively, when FACS sorts human brain-derived nuclei. This figure modified from Reference 12.

Our group and another recently identified multiple novel cryptic exon inclusion events due to the loss of TDP-43 functions in human neurons ^(12), (13)^. Our group reanalyzed valuable RNA sequencing data created by Dr. Edward Lee’s team ^(14)^. To identify changes associated with the loss of TDP-43 from the nucleus, they used fluorescence-activated cell sorting (FACS) to enrich neuronal nuclei, either with or without TDP-43. They then performed RNA sequencing to compare the transcriptomes between TDP-43+ and TDP-43- neuronal nuclei from the brains of FTD/ALS patients. As a result, they revealed a multitude of interesting differentially expressed genes ^(14)^. We reanalyze their data differently―searching for novel cryptic exon splicing events by the loss of TDP-43 using the new pipeline. As a result, we identified 66 novel cryptic splicing events in addition to *STMN2* cryptic exon. Interestingly, the *UNC13A* cryptic splicing event is one of the most significant (Figure 2) ^(12)^. Thus, we immediately paid attention to the *UNC13A* gene since it is one of the top hits for ALS/FTD in the genome-wide association studies ^(15), (16)^.

Without nuclear TDP-43, a cryptic exon of 128 bp is included in *UNC13A* mRNA, which introduces a premature stop codon. TDP-43 binding sites in the intron harbored this cryptic exon, suggesting that TDP-43 directly regulates this splicing event. We analyzed a series of postmortem samples from the Mayo Clinic Brain Bank and the New York Genome Center and found the inclusion of the novel *UNC13A* cryptic exon in the brains of FTLD-TDP and ALS patients ^(12)^. At the same time, another team also identified the cryptic exon inclusion in *UNC13A* due to TDP-43 depletion. They found this cryptic exon by detailed analysis of RNA sequencing from induced pluripotent stem cells (iPS)-derived neurons depleted for TDP-43 ^(13)^. Thus, both teams have demonstrated the cryptic splicing event in the *UNC13A* gene by TDP-43 dysfunction. This cryptic splicing event decreases normal UNC13A mRNA and protein since the abnormal *UNC13A* mRNA containing cryptic exon is degraded by nonsense-mediated decay (NMD), the mechanism to maintain protein quality ^(13)^. *UNC13A* encodes a critical neuronal protein essential for neurotransmitter release in the synaptic vesicles ^(17), (18)^. Therefore, the cryptic exon inclusion event in *UNC13A* could cause neuronal dysfunction in ALS/FTD pathogenesis.

*UNC13A* is one of the remarkable genetic risk factors for ALS and FTD, but it remains unclear how genetic variants, single nucleotide polymorphisms (SNPs), in the *UNC13A* gene increase the risk for the diseases. Notably, the two risk SNPs in *UNC13A* are located around the cryptic exon ^(12), (13)^, indicating the strong connection between the SNPs, risk for ALS/FTD, and the inclusion of cryptic exon. Figuring out how GWAS hits connect to disease pathogenesis has been a challenge. However, connecting genetics to pathology, both groups found that human brain samples derived from ALS/FTD patients harboring the risk alleles for these two GWAS SNPs have more *UNC13A* cryptic exon inclusion than those without risk alleles ^(12), (13)^. These SNPs are insufficient to cause cryptic exon splicing on their own since we did not detect them in the RNA sequencing data of healthy control samples with risk alleles ^(12)^. Instead, this genetic vulnerability is likely TDP-43-loss dependent, not causing pathogenesis until TDP-43 becomes dysfunctional.

*UNC13A* cryptic exons are abundant in the brains of patient with ALS and FTD, and the risk of SNPs in the gene potentiates the accumulation of these cryptic exons. Indeed, patients with one copy of the risk SNPs live shorter than those with zero, and those with two copies of the risk SNPs live even shorter ^(12)^. This fact is consistent with previous analyses indicating decreased survival in patients with ALS and FTD harboring *UNC13A* variants ^(19)^. Thus, since genetic variants in *UNC13A* that increase cryptic exon inclusion are associated with decreased patient survival, we speculate that therapeutic strategies to block this splicing event could have a partial therapeutic benefit. Discovering a novel TDP-43-dependent cryptic splicing event in ALS/FTD risk gene opened new provocative directions for validating *UNC13A* as a biomarker. Although we should think that this might be just a part of the whole, we now have a sensitive way of detecting the cellular consequences of TDP-43 loss of function, even before TDP-43 nuclear depletion and cytoplasmic aggregation appear. Our method will allow a more sensitive study of ALS and FTD mechanisms and pathology in brain tissues derived from patients.

## 5. TDP-43 and Other hnRNPs Regulate *UNC13A* Cryptic Splicing Event

TDP-43 belongs to the heterogeneous nuclear ribonucleoproteins (hnRNPs) family, which essentially functions to regulate multiple aspects of RNA metabolism ^(20)^. Similar to TDP-43, other hnRNPs (C, K, L, M, PTBP1) are known to suppress cryptic exon inclusion ^(21), (22), (23), (24), (25), (26)^. Several hnRNPs have been identified as regulators of the alternative splicing events of *sortilin1*, one of the TDP-43 targeting genes ^(24)^. It suggests that many proteins working in the connection within a network are critical for regulating the alternative splicing targeted by TDP-43. Therefore, this study aimed to further investigate the role of TDP-43 and other hnRNPs in regulating *UNC13A* cryptic splicing ^(27)^. The findings revealed that hnRNP L, hnRNP A1, and hnRNP A2B1 bind *UNC13A* RNA and regulate its cryptic splicing event. Higher levels of hnRNP L are associated with lower levels of *UNC13A* cryptic RNA in FTLD-TDP cases. Furthermore, when TDP-43 protein levels are depleted, hnRNP L can reduce *UNC13A* cryptic exon inclusions ^(27)^. We indicated that other hnRNPs, particularly hnRNP L, can regulate *UNC13A* splicing under loss of TDP-43 function, working as a potential disease modifier.

## 6. TDP-43 Autoregulatory Mechanism

Interestingly, TDP-43 regulates the splicing of its own *TARDBP* mRNA, encoding TDP-43, similar to other target genes. It has been found that TDP-43 strictly autoregulates the expression levels of TDP-43 in the nucleus by regulating alternative splicing in *TARDBP* mRNA as follows: In healthy status, TDP-43 binds the *TARDBP* pre-mRNA 3′UTR in the nucleus and regulates its alternative splicing ^(28), (29), (30)^. An increased level of nuclear TDP-43 results in its alternative splicing to induce the variants, which are sensitive to NMD, to reduce TDP-43 levels. Conversely, when the level of TDP-43 in the nucleus decreases, the *TARDBP* mRNA levels increase following the reduction of its alternative splicing. Therefore, *TARDBP* mRNA expression levels continually increase in ALS-affected cells with reduced nuclear TDP-43 levels ^(30)^. The formation of cytoplasmic TDP-43 aggregates is estimated to enhance the depletion of the TDP-43 level in the nucleus ^(31)^. Consequently, cytoplasmic TDP-43 aggregates cause the disease progression via the perturbation of these autoregulatory mechanisms ^(32)^.

## 7. Abnormal Splicing Variants as Potential Biomarkers

The aberrant splicing events containing cryptic exon inclusion can be considered potential biomarkers for diagnosis, prognosis, and evaluation of the efficacy of therapeutics (Figure 1) ^(8)^. Several candidate fluid biomarkers have been known, such as vascular endothelial growth factor ^(33)^ and neurofilament light chain protein ^(34)^. However, these markers reflect nonspecific neurodegeneration rather than specific pathogenesis of ALS/FTD. The next step is to evaluate these novel TDP-43-dependent abnormal splicing events and validate them in the biofluids derived from ALS and FTD patients. Since some of the cryptic exon splicing events result in the production of cryptic peptides, the levels of these peptides reflect the loss of TDP-43 function in the affected brains. Monitoring the levels of cryptic peptides in the samples derived from patients is one of the biggest challenges in the ALS and FTD field. Even though the STMN2 cryptic exon is highly abundant in the central nervous systems ^(9), (10), (11)^, detecting the cryptic peptide from truncated STMN2 has remained elusive. UNC13A and other cryptic peptides cannot be translated, owing to NMD, or are cleared immediately and thus impossible to detect ^(8)^. They may be low or not released outside the affected cells even if translated. Moreover, the success of the biomarker for cryptic peptides depends on the ability to create high-quality and specific antibodies. In this context, two independent research groups have recently demonstrated that, for some TDP-43 target splicing events translated in-frame manner, abnormal peptides from abnormal splicing variants, including cryptic exons, can be detected in human cerebrospinal fluid ^(35), (36)^. Therefore, we strongly suspect that the field will unveil new facets of ALS and FTD pathology once it develops suitable tools to detect the series of aberrant splicing events.

## 8. Conclusion and Future Perspectives

The emergence of aberrant splicing events as a disease mechanism in ALS/FTD opens up many exciting possibilities for robust novel biomarkers. One challenge may be to determine which of these dozens of new TDP‐43 splicing targets contribute to ALS/FTD. Supposing many splicing events contribute to disease simultaneously, targeting them one by one should be very difficult, and other radical approaches focused on improving the TDP-43 function itself might be needed. Indeed, there are strong connections between UNC13A, the cryptic exon inclusion event, and human genetics, letting us focus on UNC13A as a marker reflecting the pathophysiology. Nevertheless, it is essential to further investigate other candidate genes involved in the ALS/FTD pathogenesis.

## Article Information

This article is based on the study, which received the Medical Research Encouragement Prize of The Japan Medical Association in 2023.

### Conflicts of Interest

None

### Author Contributions

YK contributed to the search of previous publications and wrote the whole manuscript.
